# A Bacillus from Infectious Vulvar Disease of Cattle1Read before the ninth annual meeting of the Missouri Veterinary Medical Association, St. Louis, Mo., October 3 and 4, 1900.

**Published:** 1901-02

**Authors:** Joseph W. Parker

**Affiliations:** St. Joseph, Mo.


### A BACILLUS FROM INFECTIOUS VULVAR DISEASE OF CATTLE.[1]

By Dr. Joseph W. Parker,
ST. JOSEPH, MO.

UNDER the title "A Cattle Disease in Marshall County, Kansas,"[2] an apparently contagious pustular disease with localization chiefly on the vulvæ of cows and heifers is described (February, 1898) by Dr. Rice P. Steddom, an inspector in the bureau. In this outbreak only females were affected, and the disease certainly was not communicated by copulation. Usually after the bursting of the pustules the resulting ulcer had a tendency to slough. The mode of communication was not determined.

[1] Read before the ninth annual meeting of the Missouri Veterinary Medical Association, St. Louis, Mo., October 3 and 4, 1900.

[2] Fifteenth Annual Report of the Bureau of Animal Industry, United States Department of Agriculture.

In the fall of 1899 an outbreak of a disease similar in character occurred near Westmoreland, Kansas. Both cows and steers were affected, on the latter the ulcers being located above the ischial tuberosities, being similar to the ulcerations on the females. In both outbreaks the disease yielded readily to treatment (silver nitrate, antiseptics).

On December 14, 1899, Dr. Steddom, who also made an investigation of the latter outbreak, furnished me specimens of diseased tissue taken from the margin of the ulcers. The specimens had been taken from living animals on the preceding day and were in good condition, having been kept at a low temperature.

On the evening of December 14th I made two stab-cultures on agar-agar from internal portions of the two larger pieces of tissue (about 3 x 2 x 1.5 inches) from different animals, obtaining pure cultures from each. The organism isolated is a bacillus resembling the *bacillus typhosus*, but somewhat smaller. It develops readily at room-temperature or at 90° F. on agar-agar, bouillon, blood-serum ; also on gelatin. On agar-agar it develops a white film which spreads rapidly and uniformly over the surface ; a slight growth develops slowly along the line of puncture. The surface growth is moist, the agar-agar apparently slightly liquefied, as indicated by the touch of the platinum wire. The bacilli usually lie singly, though sometimes forming chains of five or six. Sporulation is suspected, but not demonstrated. On bouillon it forms a characteristic white film on the surface, clouding the medium within forty-eight hours. The length varies from slightly ovoid to twice the width of the bacillus. On blood-serum a white growth forms which penetrates more deeply than on agar-agar and liquefies the medium slowly. On nutrient gelatin a cup-shaped depression was formed, but if liquefaction occurred vaporization immediately followed, for no liquid was visible in the culture tube. The organism stains with a watery solution of methylene-blue or fuchsin, but best results were obtained by use of Ziehl's solution, staining one minute. In some of the specimens stained the bacilli showed unstained areas, probably indicating spore-formation.

On December 29, 1899, a rabbit was inoculated subcutaneously (behind the left shoulder) from the first culture on agar-agar. Sixty hours afterward the rabbit was found dead. Post-mortem showed inflammation and gelatinous infiltration (no pus formation) about two inches in diameter, surrounding the point of inoculation and extending throughout the thickness of the costal walls; visceral organs and blood were normal. The agar-agar cultures were made from different points in the inflamed subcutaneous tissues, as far as practicable from the point of inoculation. Both tubes developed pure cultures of the organism first isolated.

This study, while incomplete in itself, may be of help to some one else in determining the cause and nature of the disease. I am indebted to Dr. I. J. Wolf, of the Kansas City Veterinary College, for valuable assistance in the laboratory work.

